# What is the agreement between intraoperative fluoroscopy and postoperative radiographs in Bernese periacetabular osteotomy?

**DOI:** 10.1186/s12891-022-06054-6

**Published:** 2022-12-30

**Authors:** Jianping Peng, Fei Xiao, Junfeng Zhu, Chao Shen, Yang Li, Xiuguo Han, Yimin Cui, Xiaodong Chen

**Affiliations:** Department of Orthopaedics, Xinhua Hospital Affiliated to Shanghai Jiao Tong University School of Medicine, Building 8, No. 1665, Kongjiang Road, Shanghai, 200092 China

**Keywords:** Periacetabular osteotomy, Hip parameters, Agreement, Intraoperative fluoroscopy

## Abstract

**Background:**

It is important to reorient the acetabular fragment into an optimal position and version to ensure a good long-term outcome after Bernese periacetabular osteotomy (PAO). Unfortunately, the intraoperative balance between overcorrection and undercorrection remains challenging for the surgeon. The purpose of this study was to answer two questions: (1) Does the femoral head coverage measured on intraoperative fluoroscopy agree with that measured on postoperative radiography? (2) What is the reliability of intraoperative fluoroscopy in identifying hip center correction in PAO?

**Methods:**

A total of 173 patients (173 hips) who underwent PAO for developmental dysplasia of the hip (DDH) at our center from July 01, 2020, to December 31, 2020, were retrospectively reviewed. Imaging data from 111 patients (female/male, 98/13; right/left, 72/39; mean age, 28.93 years) were included in this study. The analysis included measurement of the lateral center-edge angle (LCEA), acetabular index (AI), anterior wall index (AWI), posterior wall index (PWI), extrusion index (EI), and medial offset distance (MO). These measurements were acquired from intraoperative fluoroscopic images and postoperative radiographs and compared by paired t test using SPSS (version 24.0). Significance was determined at a *p* value of < 0.05. Bland–Altman analysis, conducted using GraphPad Software (version 9), was used to quantify the agreement between intraoperative fluoroscopic images and postoperative radiographs.

**Results:**

The means (standard deviations, SDs) of the LCEA, AI, AWI, PWI, EI, and MO obtained on intraoperative fluoroscopy were 32.86° (5.73°), 0.66° (5.55), 0.29 (0.10), 0.75 (0.17), 11.15% (6.50%), and 8.49 mm (3.68 mm), respectively. On postoperative radiography, the corresponding values were 32.91° (6.31°), 1.63° (5.22°), 0.29 (0.15), 0.85 (0.14), 11.27% (7.36%), and 9.60 mm (3.79 mm). The differences in the LCEA, AWI, and EI acquired from intraoperative fluoroscopic images and postoperative radiographs were not significant (*p* = 0.90, 0.95, and 0.83, respectively), but those in the AI, PWI, and MO were significant (*p* < 0.05). The mean biases (95% limits of agreement) of the LCEA, AI, AWI, PWI, EI, and MO were − 0.04 (− 6.85), − 0.97 (− 7.78), 0 (− 0.30), − 0.11 (− 0.36), − 0.12 (− 11.92), and − 1.11 (− 5.51), respectively.

**Conclusion:**

The LCEA, EI, and AWI can be used to reliably predict postoperative femoral head coverage at the level of 2D graphics. Acetabular inclination can be cautiously assessed using AI on intraoperative fluoroscopy. In the absence of intraoperative 3D image evaluation, the AWI and PWI demonstrate acceptable agreement between fluoroscopy and radiography in assessing the acetabular version. Although the MO shows slight bias, it can be helpful in properly positioning the acetabulum during PAO.

## Background

Bernese periacetabular osteotomy (PAO), introduced by Ganz in 1988 [1], has become one of the most common surgical procedures for improving femoral head coverage by reorienting the shallow acetabulum. This procedure has been reported to relieve hip pain and improve function in patients with symptomatic dysplastic hips [2–5]. However, Bernese PAO is a complex surgical procedure with a substantial learning curve. The osteotomy is accompanied by four cuts: a complete cut of the superior pubic ramus, an incomplete cut at the ischium (as the posterior column of the innominate bone must remain intact), a cut from the anterior aspect of the iliac wing to a point approximately 1 cm superolateral to the brim of the true pelvis, and a cut connecting the first and third cuts through the posterior column.

After the osteotomy, the acetabulum can be freely rotated and mobilized, which not only improves the femoral coverage but also optimizes the rotation center of the hip joint. Although the anatomy around the acetabulum is complex and close to the iliac vessels and sciatic nerves, it is not very difficult for surgeons to complete the osteotomy with monitoring by intraoperative fluoroscopy after clearing the learning curve. However, reorienting and confirming the optimal orientation and version of the acetabular fragment [6], as well as balancing overcorrection and undercorrection [7, 8], remain challenging for surgeons. Eduardo [8] reported that the prevalence of over/undercorrection was 22% and that hips with more severe dysplasia preoperatively were at a higher risk for undercorrection, as assessed with the lateral center-edge angle (LCEA).

Intraoperative radiography or fluoroscopy, computer-assisted navigation [9], customized templates [10], and another novel device [11] have been used in Bernese PAO for judging and confirming the correction of the acetabular fragment. Of these methods, intraoperative fluoroscopy is still the most common because of its convenience, time efficiency, low cost, and low radial exposure dose. However, anteroposterior (AP) radiography and intraoperative fluoroscopy have different image acquisition protocols. Some studies have been performed to investigate the reliability of intraoperative fluoroscopy. Charles [12] reported that the intraoperative fluoroscopic assessment of PAO correction was correlated with that of postoperative radiography, but the study had a small sample size, and the correlation analysis did not represent the agreement of parameters. Another study with a larger sample confirmed the reliability and accuracy of intraoperative fluoroscopy [13]. However, the study only addressed correction of lateral coverage, as judged by the LCEA and the acetabular index (AI), and not anterior or posterior coverage of the femoral head. Additionally, neither of these previous studies discussed the accuracy of the hip center determined by intraoperative fluoroscopy.

This study was performed with the aim of answering two questions: (1) Does the femoral head coverage measured on intraoperative fluoroscopy agree with that measured on postoperative radiography? (2) What is the reliability of intraoperative fluoroscopy in identifying hip center correction in PAO?

## Methods

After receiving institutional ethics review board approval, we retrospectively reviewed the radiographs of all 173 patients (173 hips) who underwent PAO for developmental dysplasia of the hip (DDH) at our center between July 01, 2020, and December 31, 2020. As previously described [14], Bernes PAO was performed by one senior surgeon (X.C.) on all of the patients through a modified Smith–Peterson approach. No patients in this cohort underwent bilateral PAO.

**Fig. 1:**
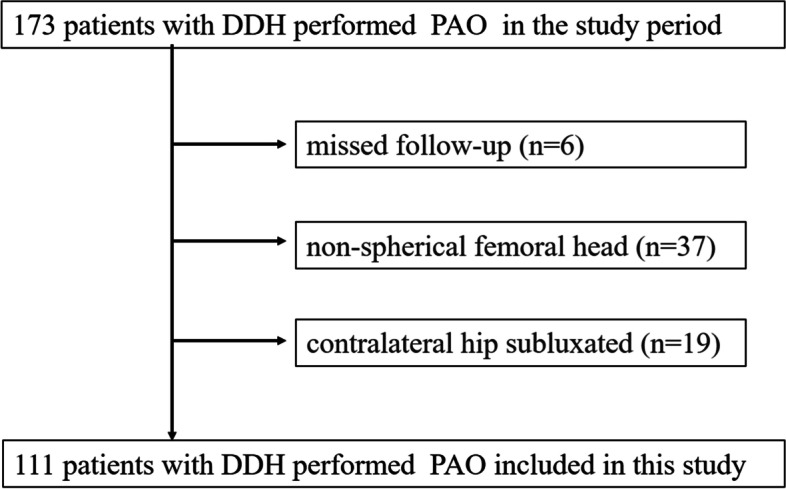
Flowchart showing patient inclusion and exclusion criteria for the study

All patients’ preoperative computed tomography (CT) images of the pelvis, images obtained by intraoperative fluoroscopy, and postoperative standing pelvic AP radiographs were obtained from our hospital’s picture archiving and communication system (PACS). The CT scanning parameters were as follows: 120 kV; 300 mAs; matrix, 512 × 512; pitch, 0.7539; field of view (FOV), 300–400 mm; and slice thickness, 0.75 mm. To eliminate pelvic tilt and rotation during surgery, we referred to Lehmann’s method [12]: the pubic symphysis must be vertical and overlying the coccyx, and the obturator foramen has a similar appearance on fluoroscopy as on preoperative standing plain pelvic radiography. Postoperative standing AP radiographs were obtained 6 months to 1 year after surgery. In all, 62 hips (62 patients) were excluded from this cohort for the following reasons: 1. previous hip surgery; 2. simultaneous proximal femoral osteotomy (PFO); 3. unavailable postoperative standing AP images of the pelvis due to missed follow-up six months to one year postoperatively; 4. significantly nonspherical femoral head, which can affect the accuracy of subsequent data measurements; and 5. marked pelvic tilt on postoperative standing radiography due to noncorrection of a subluxated contralateral hip. Finally, the imaging data from 111 patients were included in this study (Fig. 1). There were 98 females and 13 males, and the mean age was 28.93 (range: 12–54) years. Right hips were affected more frequently than left hips in the cohort (72/39).

All standing AP radiographs of the pelvis and intraoperative fluoroscopic images were obtained as per the protocols recommended in previous studies [8, 12, 15]. At our center, an intraoperative false-profile radiograph is neither routine nor necessary.

Intraoperative fluoroscopic images and postoperative standing AP radiographs of the pelvis were analyzed by an orthopedic surgeon (J.P.). The analysis included measurement of the LCEA, AI, anterior wall index (AWI), posterior wall index (PWI), extrusion index (EI), and medial offset distance (MO), as described in previous reports [16–19]. The MO was calibrated using the ratio of the femoral head diameter as measured on preoperative CT, performed for positioning, to that on intraoperative fluoroscopy and pelvic radiography.

### Statistical analysis

The LCEA, AI, AWI, PWI, EI, and MO acquired by intraoperative fluoroscopy and postoperative radiography were compared by paired t test using SPSS (version 24.0). Significance was determined at a *p* value of < 0.05. Bland–Altman analysis, conducted using GraphPad Software (version 9), was used to quantify the agreement between intraoperative fluoroscopy and postoperative radiography. The bias was estimated by calculating the mean difference and the 95% limits of agreement (LOA) between the intraoperative fluoroscopic images and the postoperative radiographs.

## Results

The results of the paired t test and the Bland–Altman analysis are shown in Table 1.

**Table 1:** Agreement between intraoperative fluoroscopy and postoperative radiograph (*N* = 111)

	Matched-Pairs T-Test				Bland-Altman Analysis		
	IN-FL	PO-RA	T value	*p*	Mean Bias	SD of bias	95% Limits of Agreement
LCEA	32.86 ± 5.73	32.91 ± 6.31	−0.13	0.90	−0.04	3.47	−6.85 ~ 6.77
AI	0.66 ± 5.55	1.63 ± 5.22	−2.93	<0.05	−0.97	3.48	−7.78 ~ 5.85
AWI	0.29 ± 0.10	0.29 ± 0.15	0.07	0.95	0.00	0.15	−0.3 ~ 0.3
PWI	0.75 ± 0.17	0.85 ± 0.14	−8.52	<0.05	−0.11	0.13	−0.36 ~ 0.15
EI	11.15 ± 6.50	11.27 ± 7.36	−0.22	0.83	−0.12	6.02	− 11.92 ~ 11.67
MO	8.49 ± 3.68	9.60 ± 3.79	−5.20	<0.05	−1.11	2.25	−5.51 ~ 3.29

*LCEA* Lateral center-edge angle, *AI* Acetabular index, *AWI* Anterior wall index, *PWI* Posterior wall index, *EI* Extrusion index, *MO* Medial offset distance, *IN-FL* Intraoperative fluoroscopy, *PO-RA* Postoperative radiograph

The means (standard deviations, SDs) of the LCEA, AI, AWI, PWI, EI, and MO obtained on intraoperative fluoroscopy were 32.86° (5.73°), 0.66° (5.55), 0.29 (0.10), 0.75 (0.17), 11.15% (6.50%), and 8.49 mm (3.68 mm), respectively. The corresponding parameters obtained on postoperative radiography were 32.91° (6.31°), 1.63° (5.22°), 0.29 (0.15°), 0.85 (0.14°), 11.27% (7.36°), and 9.60 mm (3.79 mm), respectively. According to the paired t test, the LCEA, AWI, and EI determined using the two imaging modalities were not significantly different (*p* = 0.90, 0.95, and 0.83, respectively); however, there was a significant difference in the AI, PWI, and MO between intraoperative fluoroscopy and postoperative radiography (*p* < 0.05).

**Fig. 2:**
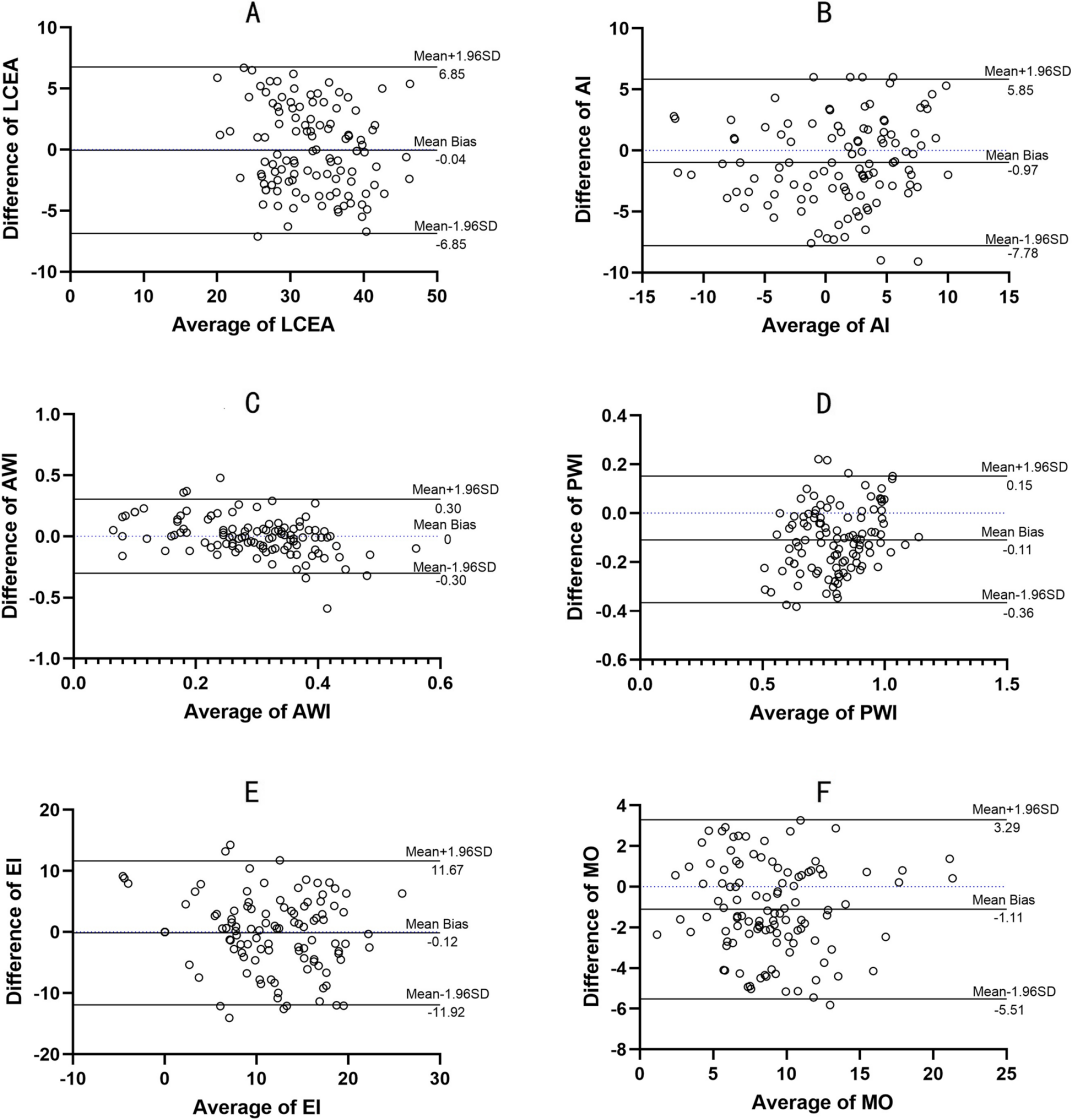
Bland–Altman plot demonstrating the mean biases in the LCEA (**A**), AI (**B**), AWI (**C**), PWI (**D**), EI (**E**), MO (**F**) between intraoperative fluoroscopy and postoperative radiography and the corresponding 95% limits of agreement

The Bland–Altman plots are shown in Fig. 2A-F. The bias and 95% LOA in the comparison between intraoperative fluoroscopy and postoperative radiography indicate that the effect of any such bias is acceptable.

## Discussion

To maximize the accuracy of intraoperative fluoroscopy, the influence of pelvic tilt and rotation needs to be eliminated during surgery. We generally confirmed the following two points: the pubic symphysis must be vertical and overlying the coccyx, and the obturator foramen has a similar appearance on fluoroscopy as on preoperative standing plain pelvic radiography. These results are consistent with the protocol reported in a previous study [12]. However, some unavoidable factors of intraoperative fluoroscopy may still create errors [20]. First, intraoperative fluoroscopy was performed with a posteroanterior beam direction. Second, the film focus distance is small. Third, the central beam is usually centered on the femoral head. Previous studies using the intraclass correlation coefficient (ICC) have shown that the measurements obtained on intraoperative fluoroscopy are correlated with those obtained on postoperative radiography [12]. However, correlation analysis cannot be used to assess the agreement between the two methods of clinical measurement [21, 22]. In this study, Bland–Altman plots were used to analyze the agreement between intraoperative fluoroscopy and postoperative radiography in assessing the outcome of PAO. The results indicated that the bias between the imaging modalities could be neglected.

This study has some limitations. First, because we excluded some patients who had undergone a previous hip surgery, who underwent simultaneous PFO, or who had a nonspherical femoral head or subluxated contralateral hip, our findings cannot be generalized to patients with these conditions. In addition, all measurements were performed by a single observer (J.P.). Since multiple prior studies have reported on interrater and intrarater reliability in measuring the LCEA, AI, AWI, PWI, EI, and MO, we did not repeat such assessments. Third, we determined that the bias of the AI, PWI, and MO as measured on fluoroscopy was acceptable based on the reference normal values reported in previous studies. Further studies are needed to determine whether the bias of these parameters affects hip function after PAO. Fourth, we did not investigate the radiograph in the supine position after the operation. However, the stability of the hip joint in the weight-bearing position is more important regarding the occurrence of pathological hip changes and symptoms. Fifth, we did not use intraoperative 3D images to evaluate femoral head coverage. Intraoperative 3D evaluation may be a more accurate method. However, this is a relatively expensive method that has not been widely used in China. Since 3D images can more accurately evaluate the coverage of the acetabulum to the femoral head, we will add postoperative 3D image data in future studies to further evaluate the accuracy and reliability of intraoperative fluoroscopy.

The LCEA of Wiberg, AI, and EI was used to assess the lateral coverage of the femoral head. Correction of the LCEA between 25° and 40°, AI between 0° and 10°, and EI ≤ 20% were defined as the target ranges after PAO based on previously published normative values [8, 23]. Charles’ study indicated that EI was less strongly correlated, with an ICC of 0.66 (0.46–0.79) [12]. Unlike the results reported by Charles, we found high agreement for the EI between intraoperative fluoroscopic images and postoperative standing AP pelvic radiographs. The LCEA and AI have demonstrated a strong correlation between intraoperative fluoroscopy and postoperative plain radiography in previous studies [12, 13]. However, a strong correlation does not imply good agreement between the two methods; correlation analysis quantifies only the degree to which two variables are related [21]. Stefanie’s [24] study indicated an acceptable agreement between the two imaging modalities using kappa statistics; however, intraoperatively, they inclined the C-arm by approximately 5° to imitate a pelvic-centered image, differing from conventional methods. In this study, the LCEA also showed high agreement between intraoperative fluoroscopic images and postoperative standing AP pelvic radiographs. In contrast to previous studies, our study found that the AI acquired by postoperative radiography was larger than that measured on intraoperative fluoroscopy (*p* < 0.05). We suspect that this difference may be due to the difficulty in determining the medial margin of the acetabular sourcil on fluoroscopic images. Charles [12] also considered fluoroscopic images to have poorer resolution than plain radiographs, potentially making it more difficult to find the necessary landmarks for measurement. Through Bland–Altman analysis, we considered this difference to be acceptable (mean bias: − 0.97°).

Proper acetabular reorientation includes not only lateral but also anterior and posterior coverage. Excessive anterior coverage is a detriment to posterior coverage and may cause impingement and adversely affect the long-term survival of the joint after PAO [6]. An anterior center-edge angle of Lequesne (ACEA), created on the false-profile view, of < 20° can be indicative of structural instability [23]. Most surgeons prefer to obtain an oblique view of the iliac crest during surgery to achieve a false-profile view. Previous studies have shown that the intraoperative ACEA is strongly correlated with that obtained on postoperative radiography, with ICCs of 0.71 (95% CI: 0.54–0.82) [12] and 0.80 (95% CI: 0.71–0.86) [15]. We chose not to measure the ACEA intraoperatively to assess the improvement in anterior coverage; although we can imitate the version of the standing pelvis by tilting the C-arm beam, we cannot simulate the version of the standing pelvis when obtaining an oblique image. Klaus [19] recommended the AWI and PWI to quantify anterior and posterior coverage. According to their report, the mean AWI and PWI were 0.41 and 0.91, respectively, for normal hips. Because these parameters for judging anterior and posterior coverage are measured on images simulating the standing pelvic version, we prefer this method to using the ACEA. In this study, the AWI obtained on intraoperative fluoroscopy strongly agreed with that obtained on postoperative radiography. Although the mean PWI obtained on intraoperative fluoroscopy was smaller than that obtained on postoperative radiography, this difference was acceptable since the mean bias was only 0.11.

A lateralized hip center is considered to be a sign of structural instability. The hip center is considered lateralized if the medial aspect of the femoral head is greater than 10 mm from the ilioischial line [25]. Medialization of the fragment could decrease the joint contact forces by decreasing the bodyweight lever arm. Troelsen [26] found that an MO distance greater than 20 mm correlated with a poor 6.8-year survivorship of PAO. Charles [20] recommended placing the medial aspect of the femoral head only 5 to 15 mm lateral to the ilioischial line. In their study, the MO showed the weakest correlation (ICC: 0.46) between measurements obtained by intraoperative fluoroscopy and postoperative AP pelvic radiography. Our data indicate that the femoral head was more medial on intraoperative fluoroscopy than on postoperative AP pelvic radiography. This difference is partly due to the different central beam positions. On the other hand, the effect of the imaging magnification ratio on the MO was more significant than that on the angle (LCEA, AI) measurements. To eliminate this effect, the MO was calibrated using the ratio of the femoral head diameter as measured on preoperative CT to that measured on intraoperative fluoroscopy and pelvic radiography. In this study, the mean bias was only − 1.11 mm, and the 95% LOA was − 5.55 mm-3.29 mm. Compared to the acceptable range of 15–20 mm [20, 26], this error is completely negligible.

## Conclusion

The LCEA, EI, and AWI can reliably predict postoperative femoral head coverage at the level of 2D graphics. The use of AI in the intraoperative assessment of acetabular inclination requires caution. In the absence of intraoperative 3D image evaluation, the agreement in the AWI and PWI between the two imaging modalities is acceptable in assessing the acetabular version. We acknowledge that it is difficult to assess the hip center position intraoperatively. Although the MO demonstrates slight bias, it can nevertheless help position the acetabulum properly during PAO.

## Data Availability

The datasets used and/or analyzed during the study are available from the corresponding author upon reasonable request.

## Abbreviations

PAO: Periacetabular osteotomy
DDH: Developmental dysplasia of the hip
LCEA: Lateral center-edge angle
AI: Acetabular index
AWI: Anterior wall index
PWI: Posterior wall index
EI: Extrusion index
MO: Medial offset distance
AP: Anteroposterior
ICC: Intraclass correlation coefficient
